# Factors important in evolutionary shaping of immunoglobulin gene loci

**DOI:** 10.1186/1745-7580-6-13

**Published:** 2010-12-06

**Authors:** Michal Barak, Guy Eilat, Ron Unger, Ramit Mehr

**Affiliations:** 1 The Mina and Everard Goodman Faculty of Life Sciences, Bar-Ilan University, Ramat-Gan 52900, Israel

## Abstract

**Background:**

The extraordinary diversity characterizing the antibody repertoire is generated by both evolution and lymphocyte development. Much of this diversity is due to the existence of immunoglobulin (Ig) variable region gene segment libraries, which were diversified during evolution and, in higher vertebrates, are used in generating the combinatorial diversity of antibody genes. The aim of the present study was to address the following questions: What evolutionary parameters affect the size and structure of gene libraries? Are the number of genes in libraries of contemporary species, and the corresponding gene locus structure, a random result of evolutionary history, or have these properties been optimized with respect to individual or population fitness? If a larger number of genes or different genome structures do not increase the fitness, then the current structure is probably optimized.

**Results:**

We used a simulation of variable region gene library evolution. We measured the effect of different parameters on gene library size and diversity, and the corresponding fitness. We found compensating relationships between parameters, which optimized Ig library size and diversity.

**Conclusions:**

We conclude that contemporary species' Ig libraries have been optimized by evolution in terms of Ig sequence lengths, the number and diversity of Ig genes, and antibody-antigen affinities.

## Background

### Evolution of V gene libraries

The adaptive immune system uses recognition molecules, the most diverse of which are the B and T cell antigen receptors that recognize specific determinants on specific antigens (Ags). When B cells are activated, their Ag receptors, also known as immunoglobulins (Igs), can be secreted as antibodies (Abs), which block and promote elimination of the Ag.

The extraordinary diversity characterizing the Ab repertoire has been generated by evolution and is then further optimized during lymphocyte development [1]. Some Variable (V) genes can provide immediate selective advantage due to their high affinity against specific conserved pathogen Ags in their germline configuration or with little modification [2]. These may have been optimized by evolution [3,4], but cannot account for the whole repertoire. Special mechanisms for creating the diversity of Abs within an individual during lymphocyte development include combinatorial rearrangement, joining imprecision, gene conversion, somatic hypermutation and the pairing of various heavy and light chains. Not all of these mechanisms are used by all vertebrate species. It has been suggested that the parameters governing Ig gene rearrangement have been optimized by evolution to maximize diversity while preventing auto-reactivity [5-8]. The diversity at the main Ag binding sites appears, however, to be encoded in the germline, and the primary role of somatic hypermutation is to extend the diversity to the surrounding regions, in order to increase fine specificity and enable the system to cope with rapidly-mutating Ags [9].

Using the advances in sequencing technologies, the V gene libraries of both the light and heavy chains have been found in numerous organisms' genomes [10-13]. In humans, V segments are classified into seven V_H_, six V_κ _and ten V_λ _families [14]. The number of different alleles of the variable Ig gene is not yet determined, as recent studies showed that there were many inaccuracies in obtaining and analyzing the data due to sequencing errors, mainly in the heavy chain [15,16]. For fish species the classification into families is less successful, probably due to high degree of intraspecies sequence divergence [11]. For example, in the rainbow trout there are at least 11 gene families of heavy chain Ig variable genes [17]. In the variable gene regions of Ig loci, two types of polymorphism are mainly observed. One is single nucleotide polymorphisms (SNP), and the other is V gene insertion/deletion polymorphisms. Half the amino acid-altering differences were observed in CDRs, despite the CDRs being much shorter than FRs, which suggests Darwinian selection [2,3]. The sizes of the Ig variable (IgV) gene libraries vary significantly between different vertebrate species, ranging from one functional IgV and 58 pseudo-IgV genes in the chicken heavy chain locus to 353 IgV genes - out of which 131 are functional - in the rat heavy chain locus [11,18].

Previous simulations of V gene library evolution focused on finding how the diversity in the genome was most probably created [19-21], and did not address the structure of gene loci and the creation of gene families. Oprea, Forrest and colleagues created a general framework to study Ab-Ag matching rules [19,21], and explored the way in which a small number of genes can create Abs that cover a large part of the Ag space. Hightower et al. showed that, when only part of the Ig gene libraries are expressed in the phenotype, there still is an evolution of fitness, but at a smaller pace [21].

The current study was aimed at addressing the following questions. (a) How do evolutionary parameters (such as the rates of various types of mutations) affect the gene locus structure? (b) Are the genomes of contemporary species a product of some optimization, i.e. is there a large parameter value space that could have generated the observed genome structures, or is it possible only in a narrow subspace? In order to understand the structure of variable gene loci and their evolution, we used a computer simulation to explore the creation of V gene libraries. We checked how the different parameters affect the size and number of gene families, as well as overall genome diversity and population fitness. The results of the simulation showed that contemporary species Ig libraries have probably been optimized by evolution in terms of Ig sequence lengths, the number and diversity of Ig genes, and the affinity (binding threshold) between the antibody and antigen.

## Methods

### Organism

**Figure 1:**
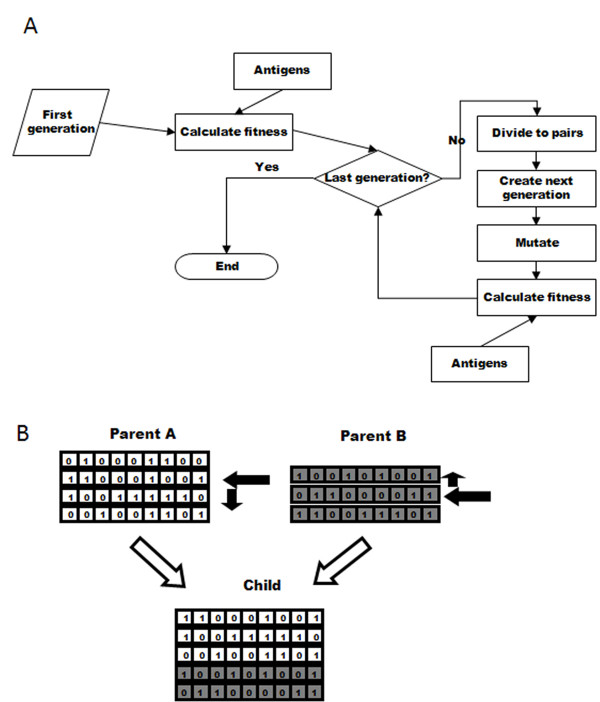
**Simulation flow and cross-over**. Schematic representation of (A) the flow of the simulation, and (B) the crossover process used to create a child's genome from its parents' genomes. In (B), boxes containing "0" or "1" represent the bits in the bit string. The arrows indicate the random choices done: selecting each parent to get the genome from, in each parent selecting the crossover start string and also selecting the direction for getting the strings in the parent genome.

The algorithm of our simulation is shown in Figure 1A. The model's basic unit is the organism, of which there is a fixed, configurable number in the simulation. Every organism has a "genome", represented by a collection of fixed-length bit strings, which represent its Ab V genes. The number of strings in an organism's genome is at least one, and the maximum number can be either unlimited, or limited by reducing the organism's fitness as a function that increases with the number of strings, representing the cost of maintaining these genes, as described below. The life span of each organism is one generation of the simulation.

### Fitness

The fitness of the organisms is based on their ability to survive the Ags they are exposed to, and determine their ability to have descendants. In every generation, the organisms' fitness values are determined as follows: A subset of strings from the genome is selected to be "expressed" and becomes the organism's phenotype, that is, its Ab repertoire. The size of the subset is determined by the Phenotype/Genotype ratio parameter and the strings are chosen randomly. This Ab repertoire is exposed to an Ag library. The Ag library is recreated every generation, with randomly generated strings, to account for the much faster evolution of pathogens relative to vertebrates, and is composed of random bit strings in the same length as the Ab bit strings. The number of Ags is configurable. Every Ab in the repertoire is checked against every Ag in the Ag library. The match is done by a simple bit comparison (using a global alignment for the comparison has resulted in non-realistic running times for the simulation). If the match between an Ab and an Ag exceeds a specified threshold, then this Ab can defend the organism from this Ag. The organism's overall fitness is calculated by summing up the maximum match values that exceeded the threshold for each Ag, and dividing this sum by the Ag library size. Thus, both the number of Abs that are over the threshold and the maximum value over it are important, and both are used in the fitness calculation. The fitness is averaged on all the Ags, so having a great protection against some Ag or good protection against more Ags has the same effect on the organism likelihood to have descendants. There is no threshold for the fitness needed for creating offspring; instead, this is determined by competition - the parents with higher fitness generate offspring before parents with lower fitness, until there is no more room in the population, such that the parents with lowest fitness do not get to reproduce. If none of the organisms survived any of the antigens, then the next generation is not created and the simulation ends.

### Creating the next generation

After the fitness is calculated for all organisms, the organisms are divided into pairs. The pairs are chosen according to the organisms' fitness: the higher the fitness, the more likely the organism will be selected to have descendents. The organism with the highest fitness is paired with the second highest, the third with the fourth and so on. The number of descendents of a pair is proportional to their combined fitness. The total number of descendents is fixed to preserve the population size, unless none of the organisms survived (all organisms have exactly zero fitness) - an unlikely outcome that would terminate the simulation. We preferred this way of representing evolutionary pressure to selecting totally random pairs, which resulted in simulation in which the average fitness did not converge, and to the more conventional procedure of selecting pairs by a biased selection in proportion to their to the fitness, as this resulted in the same qualitative results as when using our selecting method, but with more fluctuations.

### Genetic Crossover

To create a new organism, a random number of genes is taken from each parent, and combined by a "genetic crossover" to create the organism's new gene library, as follows (Figure 1B). A gene is chosen randomly in each parent, with a normal probability distribution around the middle of the gene library. The chosen gene is then included in the child's genome, along with all the genes that are either upstream or downstream from it in the parent library (the direction is chosen randomly). The choice of which parent will be used first is also random. The resulting genome size is thus at least one bit string, and its size can differ from the parents' library sizes. As a result of this process gene locations in the child's library can differ from their location in the parents' libraries.

### Mutations

After a new organism is created, its genome has a chance of undergoing one or more mutations. The rate for each of the following mutation types can be set separately.

• A point mutation that changes a randomly chosen bit in a randomly chosen gene (bit string) in the organism's gene library.

• In a duplication, a randomly chosen gene from the genome is duplicated, and the new copy is inserted into the genome in a randomly chosen place, with a high probability to be near the original gene.

• In a deletion mutation, a randomly chosen gene is deleted from the genome and the new genome size is thus reduced by one.

• An exchange mutation swaps the locations of two randomly chosen genes.

• The last type of mutation is deleting a randomly chosen bit from a randomly chosen gene, inserting as the last bit a new bit with the same value as the deleted bit's value, to preserve gene size.

After creating all the offspring, the system is ready for the next cycle.

### Repertoire characterization

After every generation, the simulation records the maximum and average organism fitness and genome size. Once every ten generations, the average diversity, calculated from individual diversities of all organisms, is recorded. For a single organism, the diversity is defined as the average distance between every two different genes. For a pair of genes, the distance is one minus the sum of the identical bits in the strings, divided by the string length. The diversity thus takes a value between 0 and 1, where 0 is for a population in which all genes are identical, and 1 is the value in the case in which for every organism, the genes in the gene library are complementary (i.e. there are only two genes in each organism which are mirror images of each other). When the diversity is 0.5, the average organism has genes in its library that have random bit string distances - 0.5. In addition, the genomes of all the organisms are recorded in the last generation.

### Gene locus structure analysis

The analysis of the genome in the last generation starts with finding the families in the genome of every organism, in two ways. The first, intended to identify only very similar family members, is to calculate the above-defined distances between the organism's genes. A distance of less than 0.2 between two sequences (equivalent to at least 80% homology) places them in the same family [22]. The second way is to use a global alignment algorithm when comparing the sequences, and to include in the same family sequences whose distance obtained from global alignment is under 0.2. Family sizes are expected to be larger when using the second method, as the global alignment algorithm increases the chance of finding similarity between sequences.

After finding the families, the following data on them are gathered. For all organisms, we find the number of families, the average and median family size (number of family members), the average genomic span of a family (the number of sequences in the genome between the first and last sequences that belong to the family), the average family size ratio - the genomic span of a family divided by the number of sequences in the genome, and the mixing index (the average of the distances between sequences in a family divided by the family size). The mixing index was thus defined, so that in a family that is not mixed at all, it will be less than 1; if it is larger than 1, it means that quite a few distances between genes in the family are larger than the family size, which can only happen if this family is mixed with other families.

The above-defined gene locus properties were studied as function of the simulation's parameters (Table 1). At the first stage, we only varied the basic simulation parameters (such as gene size or the number of Ags), and examined their effects on fitness and genome diversity. These initial results were used to set the basic parameter default values. Then we proceeded to examine how the various mutation rates affect all the measured gene locus characteristics.

**Table 1:** Simulation parameters

Parameter	Description	Default value	Range
**Basic Parameters**			

Generations:	The number of generations in the simulation	20000	

Population size:	The number of organisms in the simulation	100	10-100

Segment length:	The number of bits in a gene segment string and in an antigen string	128	128, 512,1024

Maximum number of segments:	The upper limit on the size of an individual's genome library	250	50-250

Binding threshold:	The minimum binding affinity between antigen and antibody that is needed for protecting the individual from the antigen	0.53	0.3-0.7

Antigen number:	The antigen library size created in every generation	100	10-1000

Phenotype/Genotype ratio	The ratio between the number of antibody segments expressed in the phenotype and their number in the genome	0.2	0.1-1

Penalty function:	Penalty added to an organism's fitness as a function of genome size	fitness/(1 + tan(π*n/(2*n_max_)) where n is the number of segments in the organism's library and n_max _is the maximum number of segments allowed.	

**Mutations Rates**	All rates are in units of events/gene/generation, except the point mutation and bit deletion rates, which are in units of events/bit/generation.		

Point mutation	The probability for every bit in every segment to change its value.	0.001	0.001 -0.014

Duplication	The probability of every segment to be duplicated	0.001	0.001 -0.014

Deletion	The probability of every segment to be deleted	0.001	0.001 -0.014

Exchange	The probability of a segment to swap its location with other randomly chosen segment.	0.001	0.001 -0.014

Inversion	The probability of a segment to change its orientation in the genome	0.001	0.001 -0.014

Bit deletion	The probability for every bit in every segment to be deleted	0.001	0.001 -0.014

The parameters used in the simulation and their default values. For value justification see text.

## Results

### Setting the default simulation parameters

We first performed an initial exploration of the parameter space (results not shown), varying all parameter values simultaneously, and based on those results, we narrowed the default value ranges of the parameters. (The simulation parameters are summarized in Table 1.) Then, we proceeded to explore each of the simulation parameters further.

### Number of generations and population size

**Figure 2:**
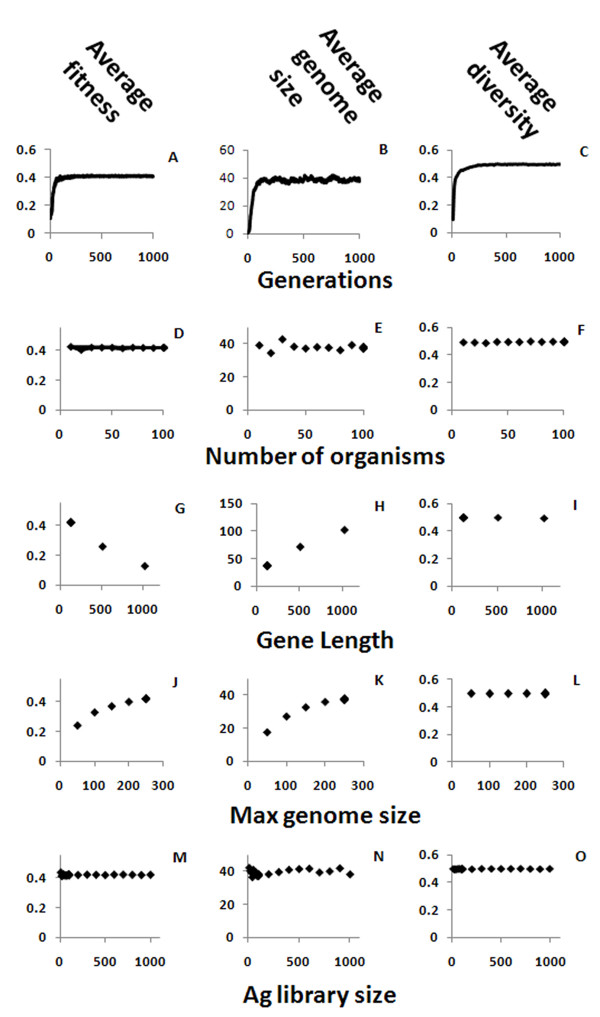
**Effects of basic simulation parameters**. The average fitness - the ability of organism to survive the Ag it exposed to (A, D, G, J, M), genome size (B, E, H, K, N) and diversity of the genes in an organism (C, F, I, L, O) of the population, are shown here as function of the number of generations (A, B, C), the gene length (G, H, I), the number of organisms (D, E, F), the maximum genome size (J, K, L) and the antigen library size (M, N, O).

The first parameter to be examined was the number of generations needed to achieve the system's steady state. This steady state is not necessarily a fixed state, as there are ongoing fluctuations in the population as it continues to evolve. However, the amplitude of these fluctuations is relatively small and the system stabilizes around a single state (that is, more or less constant average values of fitness, genome size and diversity) after a relatively small number of generations - around 500. The number of generations in the simulation was set to 20,000, or until all the organisms have perished. The latter condition was rarely encountered and used only in extreme conditions. The 20,000-generation limit was high enough so that the effect of the initial conditions was small and the system quickly reached equilibrium (Figure 2 A-C). The genome size under the default parameters has settled to an average of about 40, which is within the range of the known Ig genomes as described above [10-18], and the average diversity approaches 0.5, which is the maximum diversity possible for random bit strings.

The size of the population in the simulations had only a small impact on the results. As the number of organisms is increased, the variability in the results is reduced, but the overall system behavior remains the same (Figure 2 D-F).

### Gene length and number

The gene length in the simulation has a fixed value. We used a size of 128 bits in most runs, but also checked the values 512 and 1,024. The effect of increasing the gene length is that, as the length is increased, fewer bits are identical in a bit match between any two genes. For example, in 128 bit-long sequences the random matching is 64 bits and for a threshold of 0.53 (the binding threshold, described in detail below, is the minimum number of identical bits needed for Ab-Ag binding) there are on average about 4 unmatched bits for totally random genes that need to be mutated in order to get an antibody string that would pass the binding threshold (128*(0.53 - 0.5)), while for sequence with 1024 bits there are about 31 unmatched bits for the same threshold (1024*(0.53 - 0.5)) that would have to be mutated. To compensate for this, more antibody genes are needed in order to cover the antigen space, and thus the genome size is increased, although it remains within the range of the known Ig genomes as described above [10-18]. Although the repertoire quickly reaches the maximum diversity of 0.5, where all the sequences are the least similar to each other (Figure 2I), genome size still increases considerably with sequence length (Figure 2H). The large number of sequences needed for covering all the possibilities when gene length is increased is probably the reason why Ig sequences in real genomes are relatively short compared to the full length of the original antigen proteins, and antibodies only bind to small epitopes on the antigens. This way, a smaller number of genes can cover a larger fraction of all possible antigens, and the required matching does not need to be perfect, but only above a binding threshold. Both effects of gene length - large genome sizes and poor bit matching - explain why the average fitness decreases as gene size is increased (Figure 2G).

The maximum number of genes in an organism (maximum genome size), used within a penalty function that reduces the fitness as a function of gene number, helps the simulation avoid the trivial but biologically incorrect solution, where all or most of the possible genes are included in the organism's genome. The penalty function reduces the fitness of an organism when the number of genes in its genome increases. We used the value of 250 genes, as it is in upper range observed in real species [10-18], and any increase above this value seemed to increase the fitness by a negligible amount. Increasing the maximum number of genes increased the genome size (Figure 2 K) until a new balance between the fitness (Figure 2 J) gained by the larger genome size and lost by the penalty was reached. This parameter did not affect the diversity of the population (Figure 2 L), as it was already close to the maximum for smaller genome sizes.

### Ag library size and binding threshold

The Ag library size has a negligible effect on overall system behaviour (Figure 2M-O). As the library is recreated randomly in every generation, under the very gross approximation that most pathogens evolve faster than organisms, the evolution of Abs acts to increase the diversity and not to achieve specificity against a static Ag.

**Figure 3:**
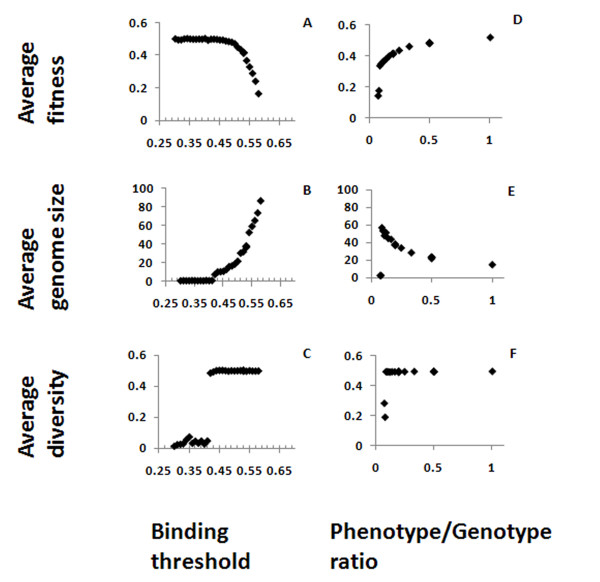
**Effects of binding threshold and phenotype/genotype ratio**. Average fitness (A, D), genome size (B, E) and diversity (C, F) are shown here as function of the binding threshold between Ab and Ag (A, B, C) or the phenotype/genotype ratio (D, E, F).

The binding threshold is the minimum fraction of bits in the Ab string that must be identical to the Ag string in order to protect the organism against the Ag. When comparing two random bit strings, 0.5 of the bits are identical on average. Hence a binding threshold lower than 0.5 would have resulted in a high organism fitness with almost no dependency on other parameters, since with such a threshold even a small number of genes - actually, one gene - has a high probability of protecting the organism against most Ags (Figure 3A). As the threshold approaches 0.5, genome size and diversity begin to rise (Figure 3B, D), since the chance of matching all Ag genes is lower for any given Ab gene. At threshold values of 0.42 and higher the genome size is increased, to compensate for this difficulty in binding and achieve a higher fitness. In addition, there is a large "jump" in the diversity due to the increase in genome size - as one random antibody gene is no longer enough to protect the organism against all antigens. When the binding threshold approaches 0.5, the fitness begins to drop; at threshold values of over 0.5 (the random match), even those compensations are not enough and the fitness is reduced; and when the threshold exceeds 0.58, the population usually dies out. We chose the value of 0.53 as a default for subsequent runs, as it is higher than the random match - which would have been unrealistic, as real antibodies must be selected not to match the "self" - and hence must be a little more specific than just a generally "sticky" molecule - without also losing their match to most Ag sequences, so they cannot be too specific. However, the value of 0.53 is not too small, so the fitness genome size and diversity are still acceptable.

### Genotype/phenotype ratio

The parameter Phenotype/Genotype ratio gives the fraction of genes in the genome that are expressed in the Ab repertoire, and was varied using the values 1, 1/2, 1/3... down to 1/10. This parameter strongly influences fitness and genome size (Figure 3 D, E). Decreasing the ratio causes a decrease in the fitness and a compensating increase in genome size. There is a limit to the compensation that can be reached by increasing the genome size, because of the limitation on the genome size in the simulation. When the Phenotype/Genotype ratio is small enough, the compensation limit is reached and, since increasing the genome does not increase the fitness anymore, the genome size and fitness drop dramatically, together with the diversity (Figure 3D-F). It is difficult to compare the ratios we used to what happens in real organisms, as complete repertoire characterizations do not exist for most of them. In zebrafish, it was found that between 50 and 86% of all possible VDJ combinations are used, and different individual fish shared a similar frequency distribution [23]. From the graphs presented in the latter study, it can be seen that some individual V genes are rarely represented in the actual repertoire, while others are more common. Similar results were found in sequencing the repertoires of a few human subjects [24]. In both cases, a fraction of the repertoire is not represented, but it does not seem to be higher than 50% in any individual, and in most cases it is must smaller - i.e., the genotype/phenotype ration in real systems is in the range that our simulations show to be "safe" for the individual.

After exploring the impact of basic parameter values (Figure 3), we chose the default values for the simulation (Table 1). Using those values, we explored the effects of changing the various mutation rates.

### Effects of Mutation rates

**Figure 4:**
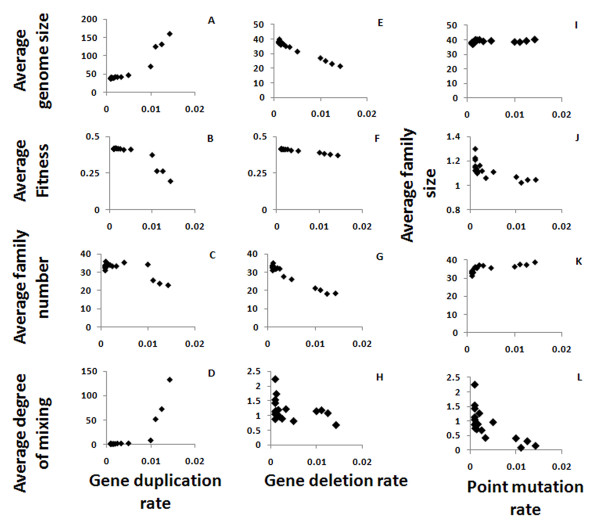
**Gene locus properties as function of the mutation rates**. Shown are the effects of segment duplication (A-D), segment deletion (E-H) and point mutation (I-L) rates on the average genome size (A, E, I), the average fitness (B, F), the average number of families in a genome of an organism (where strings which have more than 80% similarity belong to the same family; C, G, K) and the average inter-mixing of the families in the genome (D, H, L). Varying the point mutation rate generated no meaningful changes in the fitness (not shown). The average family size - which is the genome size divided by the family number - is shown as function of the point mutation rate (J) to illustrate the rapid drop in the family size as the mutation rate increases. The size of the genome is increase drastically as a function of the segment duplication rate, and so does the mixing of the families (note the different scales).

The rates of occurrence of different mutation types were varied between simulation runs (Table 1), and their effects on average fitness, genome size, diversity, and gene locus structure were examined. Some of the results are intuitively obvious, e.g., that increasing the gene duplication rate increases the average genome size (Figure 4A). Others are less obvious and several properties are influenced by several different mutation rates, as described in the following sections. In the results presented below, global alignment was used to find the members of a family, and the average over all organisms is shown for each gene locus property. The ranges of mutation rates we used cover the transition from the region where the impact of the mutation is negligible and its effects are controlled by other factors in the system, to the region where the mutations dominate the system's behaviour - as in the case of duplication mutations increasing the genome size uncontrollably.

### Gene duplication rate

The gene duplication rate directly affects the genome size. However, this effect is significant only when the duplication rate is high enough - over 0.01 events per gene per generation, which can also be considered very high in terms of real systems (Figure 4A). Genome size, limited by the maximum genome size and by the Phenotype/Genotype ratio, has an optimal value, and the larger genomes created by higher duplication rates are corrected to the preferred size. However, when the duplication rate is high enough, the genome size still increases and so the fitness decreases (Figure 4B). The number of families in the genome - which is between 10 and 40 in our simulations, as in real genomes - decreases when the duplication rate increases (Figure 4C), again because overall genome size is limited. The degree of inter-family mixing (the average radius of the family divided by its size) increases with the gene duplication rate, as the frequent duplication of genes that belong to the same family but are separated by members of other families increases the mixing (Figure 4D), up to unrealistic values of >100 (that is, distances of 100 genes or more between genes from the same family) for the highest values of the gene duplication rates.

### Gene deletion rate

The same set of optimization mechanisms that play a role in the case of gene duplication is also at work in the case of gene deletion. When the gene deletion rate is low, the average genome size converges to the optimal size, but it decreases if the deletion rate is too high (Figure 4E). Unlike in duplication, the fitness decline rate as function of gene deletion rate is negligible (Figure 4F). However, similarly to the effect of changing the duplication rate, the number of families decreases as the gene deletion rate is increased (Figure 4G). The degree of mixing is only high (>2 - which is the value we'd expect to get in real genomes) for very low segment deletion rates, and for higher values it decreases, as expected since deletion contradicts the mixing effects caused by gene duplications (Figure 4H).

### Point mutation rate

The point mutation rate has a negligible effect on genome size (Figure 4I). The average family size decreases slightly and family number increases slightly with an increase in the point mutation rate, which is expected since the mutations decrease the similarity between genes and hence the gene families are smaller (Figure 4J, K). These changes are very small compare to the changes caused by gene deletion and duplication. Since families become smaller when the point mutation rate is higher, the mixing between them is also reduced (Figure 4L), similar to the effect of increasing the point mutation rates.

## Discussion

The human Ig variable region gene locus has undergone extensive evolutionary editing. This can be seen by the division to families, where every family probably started from a single gene that was duplicated and mutated to form sets of similar but not identical sequences. The aim of the current study was to answer the following questions. What evolutionary parameters affect the size and structure of gene libraries? Are the numbers of genes in libraries of contemporary species, and the corresponding gene locus structure, a random result of evolutionary history, or have these properties been optimized with respect to individual or population fitness? To aid us in answering those questions, we created a simulation of the evolution of Ig gene libraries in a population of organisms.

Although it is difficult to directly relate rates and other simulation parameters to actual evolutionary rates, we may examine the general characteristics of real Ig gene loci in order to draw conclusions regarding whether the current Ig variable gene loci have been optimized in any way. Our results suggest that the Ig gene locus structure has been optimized by evolution, based on the following observations. Our simulations show that the population of organisms, after a stochastic start, settles into to a steady state with the maximal fitness that can be reached within the limiting conditions of the chosen parameters. This is achieved by evolution towards the optimal combination of genome size and diversity for the given set of parameters. This optimization is not observed when the size of the genome is unlimited and the organisms' genomes can grow indefinitely. As the Ig gene loci of contemporary organisms do have limited sizes, it is reasonable to conclude that there is a maintenance cost that limits the number of Ig gene segments. If there was no limitation on gene locus size, we would have expected that large numbers of genes will have an evolutionary advantage. This advantage would have manifested itself in the largest possible number of genes appearing in all species. However, there are large differences in the number of genes between species [11]. For example, in the chicken genome, where the main diversity mechanism is gene conversion, only one functional V gene for each of the light and the heavy chains is found in the genome [25,26], and the fish *Raja erinacea *has no somatic diversification and therefore has a larger germline Ig gene diversity with amino acid sequence differences preferentially distributed in complementarity-determining versus framework regions [27]. Additionally, the rat genome has a 353 IgV genes in the heavy chain (IgVH) while humans and zebrafish have much lower numbers of IgVH genes (104 and 47, respectively). As the genes are created by gene duplications, if there were no limiting mechanisms, we would also expect a smaller degree of diversity in the genes. However, the data show that more evolutionarily advanced species do not necessarily have more genes, and the genes have large diversity [11]. Preference for diversity is found in the rabbit, where the polymorphism in the dominant V_H _is highly conserved through [28,29]. Sharks also show a selection aimed at increasing the V_H _diversity [30]. Ig locus sizes and arrangement in different species may reflect the different diversity-generating processes they use, for example translocon organization (locus with multiple V and few or one C genes) versus clusters (multiple loci each containing one to three V and one C genes) [31].

Our simulations show that the Phenotype/Genotype ratio has an optimal range, below which the fitness is too low, as the benefits of having a large gene library to creates a large enough Ab repertoire are counteracted by the overhead of maintaining a large gene library (Figure 3), and above which the fitness is not significantly increased.

Human IgH variable region gene loci contain up to ten gene families, with a total number of genes of at most 123, the diversity of which is quite high. Together with the diversity of the IgL and with the additional mechanisms of diversity generation not modeled here, such as gene rearrangement and junctional diversity, the Ab repertoire generated in normal individuals, although it covers only a small fraction of potential receptors, is evidently sufficient for survival. That is, the Phenotype/Genotype ratio is probably in the range that allows reasonable diversity and hence fitness, and also a reasonable gene locus size. Comparing our results to the repertoire coverage in those species for which repertoire sequencing for single individuals has been performed, i.e., fish [23] and humans [24], we see that the fraction of V genes used is within the range that our simulations identify as "safe" for the individual.

The binding threshold in the simulation must be more than the random 0.5 match, to prevent antibodies from being so "sticky" as to be potentially autoreactive while minimizing the loss of matching to possible foreign Ag sequences. On the other hand, a too-large threshold results in a too-small coverage of the Ag sequence space. Values should be in the order of 0.55, as for higher values the coverage becomes too small, and the organisms become extinct. This result is in agreement with recent studies showing that low-avidity interactions between an Ag and the B-cell receptor can induce deletion, receptor editing and T-dependent immune responses, suggesting that high-avidity binding of the Ag is not essential [9]. As actual Igs are not bit strings and the matching to the Ag is through amino acids with a 3-dimensional structure, the optimal matching value and length that are small enough to cover the maximum Ag space and large enough to implement self-tolerance without compromising Ag recognition, as evidenced by the relatively small size of antibody binding epitope size relative to the total antigen size.

Our simulation also shows that the gene length should not be too large, as the possible number of Ag genes increases exponentially with gene length. Keeping antibody genes short, as observed in real species genomes (relative to the full antigen size as explained above), together with the relatively low binding threshold, allows the Ab repertoire to cover a large portion of the Ag space and thus aids in the survival of the organisms. As the number of human Ig variable region genes is by no means the largest observed, we may also conclude that the gene duplication and deletion rates were evolutionarily optimized to a range that would not cause the decrease in fitness shown by our simulation.

When gene deletion or duplication changes the genome size, the evolutionary process can restore the gene library size back to its optimal size, and so maintain the fitness values. If the rate of gene deletion or duplication is too high, our simulation shows that evolution cannot restore the library size to the optimal one in a way that will maintain the organism population's fitness. From this we conclude that the range of rates of Ig gene deletion and insertion were also optimized during evolution, and organisms with too-high rates became extinct because of their reduced fitness.

While V(D)J recombination is an important contributor to V region diversity, it is out of the scope of the model presented here, which deals only with the evolution of V segments, for the following reasons. (a) Evolutionary changes in the IG gene locus, such as duplications and mutations, operate on segments and not on the recombined genes. (b) Most of the length of the variable region gene is due to the length of the V segment, hence the insights gained for the length of the V segment should be applicable for the whole gene. (c) Most of the inheritable variability in Ig genes is contained within the V segments; while much variability is added by recombination, it is not inheritable. Additionally, most of the binding to the Ag is done by the longer V genes and not the D and J genes, so we focused our study on them, but the same principles that govern V gene evolution should also apply to the evolution of the D and J genes. Evolution has reduced the cost of maintaining large segment libraries by generating diversity through recombination. However the V segment libraries are still large, and their structures - i.e., the composition of the families and in the order the family members appear in the genome - are extremely diverse in the different vertebrates [10,11] and thus their evolutionary optimization is still an interesting question.

The structure of the families of IgV gene is extremely diverse in the different vertebrates; both the composition of the families and in the order the family members appear in the genome vary considerably. As our simulation does not include the somatic diversity, our results should be more relevant to species that use genomic diversity as the main diversity generator. Indeed, in the simulation results (Figure 4) for most cases the size of families is close to 1, similar to the repertoires found in fish species that rely mostly on genomic diversity, and have a small similarity between genes [11].

## Conclusions

Overall, we may conclude that contemporary species' Ig gene libraries were optimized by evolution. We believe that the fact that not all parameter value combinations yield acceptable results in and of itself shows that some optimization of the IgV loci has occurred during evolution. The parameters to which the results are most sensitive are the binding threshold between Ab and Ag, the length of the Ig gene, and the genome size. The gene length is optimized so that, on one hand, it would not be too short, which would make the selection against self reactivity almost impossible. On the other hand, gene length would not be too long, so that the Ag space can be mostly covered. The number of Ig gene genes is optimized so that the cost of maintaining it will not be too high, and the diversity will not be too low. The affinity threshold (binding threshold) between the Ab and the Ag is optimized so that self-tolerance will be possible while creating a maximum diversity of Igs. Diversity of the Ig genes, combined with the Ig library size, is thus sufficient to protect the organisms from almost all possible Ag.

## List of abbreviations

Ab: Antibody; Ag: Antigen; CDRs: Complementarity determining regions; FRs: framework regions; Ig: immunoglobulin; V: Variable region gene.

## Competing interests

The authors declare that they have no competing interests.

## Authors' contributions

The study was designed by RM and performed by MB; GE has made several improvements to the program; and RU has advised on the algorithm. The paper was written primarily by MB with additions by RM and RU. All authors read and approved the final manuscript.
